# Local Social Vulnerability as a Predictor for Cancer-Related Mortality Among US Counties

**DOI:** 10.1093/oncolo/oyad176

**Published:** 2023-06-19

**Authors:** Krista Y Chen, Amanda L Blackford, Ramy Sedhom, Arjun Gupta, S M Qasim Hussaini

**Affiliations:** Johns Hopkins School of Medicine, Baltimore, MD, USA; Sidney Kimmel Comprehensive Cancer Center, Johns Hopkins Hospital, Baltimore, MD, USA; University of Pennsylvania, Philadelphia, PA, USA; Masonic Cancer Center, University of Minnesota, Minneapolis, MI, USA; Sidney Kimmel Comprehensive Cancer Center, Johns Hopkins Hospital, Baltimore, MD, USA

**Keywords:** county, rural, cancer, disparities, policy

## Abstract

Substantial gaps in national healthcare spending and disparities in cancer mortality rates are noted across counties in the US. In this cross-sectional analysis, we investigated whether differences in local county-level social vulnerability impacts cancer-related mortality. We linked county-level age-adjusted mortality rates (AAMR) from the Centers for Disease Control and Prevention (CDC) Wide-ranging Online Data for Epidemiologic Research database, to county-level Social Vulnerability Index (SVI) from the CDC Agency for Toxic Substances and Disease Registry. SVI is a metric comprising 15 social factors including socioeconomic status, household composition and disability, minority status and language, and housing type and transportation. AAMRs were compared between least and most vulnerable counties using robust linear regression models. There were 4 107 273 deaths with an overall AAMR of 173 per 100 000 individuals. Highest AAMRs were noted in older adults, men, non-Hispanic Black individuals, and rural and Southern counties. Highest mortality risk increases between least and most vulnerable counties were noted in Southern and rural counties, individuals aged 45-65, and lung and colorectal cancers, suggesting that these groups may face highest risk for health inequity. These findings inform ongoing deliberations in public health policy at the state and federal level and encourage increased investment into socially disadvantaged counties.

## Teaser

Gaps in national healthcare spending and disparities in cancer mortality rates are noted across counties in the US. This study investigated whether differences in local county-level social vulnerability impacts cancer-related mortality.

## Background

An individual’s lived social and economic environment, collectively known as social determinants of health, impacts access to timely care in a disease with complex treatment planning, high costs, and great social impact.^1^ Adverse social determinants of health may predispose underserved communities to poor cancer outcomes including delayed diagnosis and lower rates of long-term survival. Across the 3143 US counties, there is great variation in social, economic, and healthcare infrastructure, varied levels of healthcare spending, and substantial differences in cancer-related mortality. Local social vulnerability may serve a key metric in exploring health inequities as it encompasses multiple social determinants of health and presents a key leverage point for federal- and state-level policy efforts to improve access to care. Here, we investigated the impact of county-level social vulnerability on age-adjusted cancer mortality rates (AAMRs).

## Methods

We linked all cancer-related deaths across US counties from 2013 to 2019 [ICD C00-C97] in the Centers for Disease Control and Prevention (CDC) Wide-ranging Online Data for Epidemiologic Research (WONDER) database to county-level Social Vulnerability Index (SVI) data from the CDC Agency for Toxic Substances and Disease Registry (ATSDR).^2,3^ CDC/ATSDR calculates SVI based on 15 social determinants of health attributes from the American Community Survey, and reports scores for overall SVI as well as 4 major subcomponents (socioeconomic status; household composition and disability; minority status and language; housing type and transportation) (Supplementary Table S1). As minority status is already included as a subcomponent and may impact mortality estimates when further comparing across racial/ethnic subgroups, a modified SVI was generated by removing minority status (all persons except non-Hispanic White adults).^4^

SVI was presented as percentile rankings by county and classified into quartiles based on their distribution among all US counties (1st [least vulnerable] = 0-0.25; 4th [most vulnerable] = 0.7--1.00). AAMRs per 100 000 individuals for all US counties were compared between 1st and 4th quartiles for overall SVI and for each of the subcomponents across demographic subgroups based on age, sex, race/ethnicity, urban-rural status, US region (Supplementary Table S2), and cancer type. Comparisons were made using robust linear regression models with a log scale and including the county population size as weights and reported as rate ratios (RR) with associated 95% CIs; CIs not including 1 were considered statistically significant. All analyses were done in R version 4.1.3.

## Results

**Figure 1.**
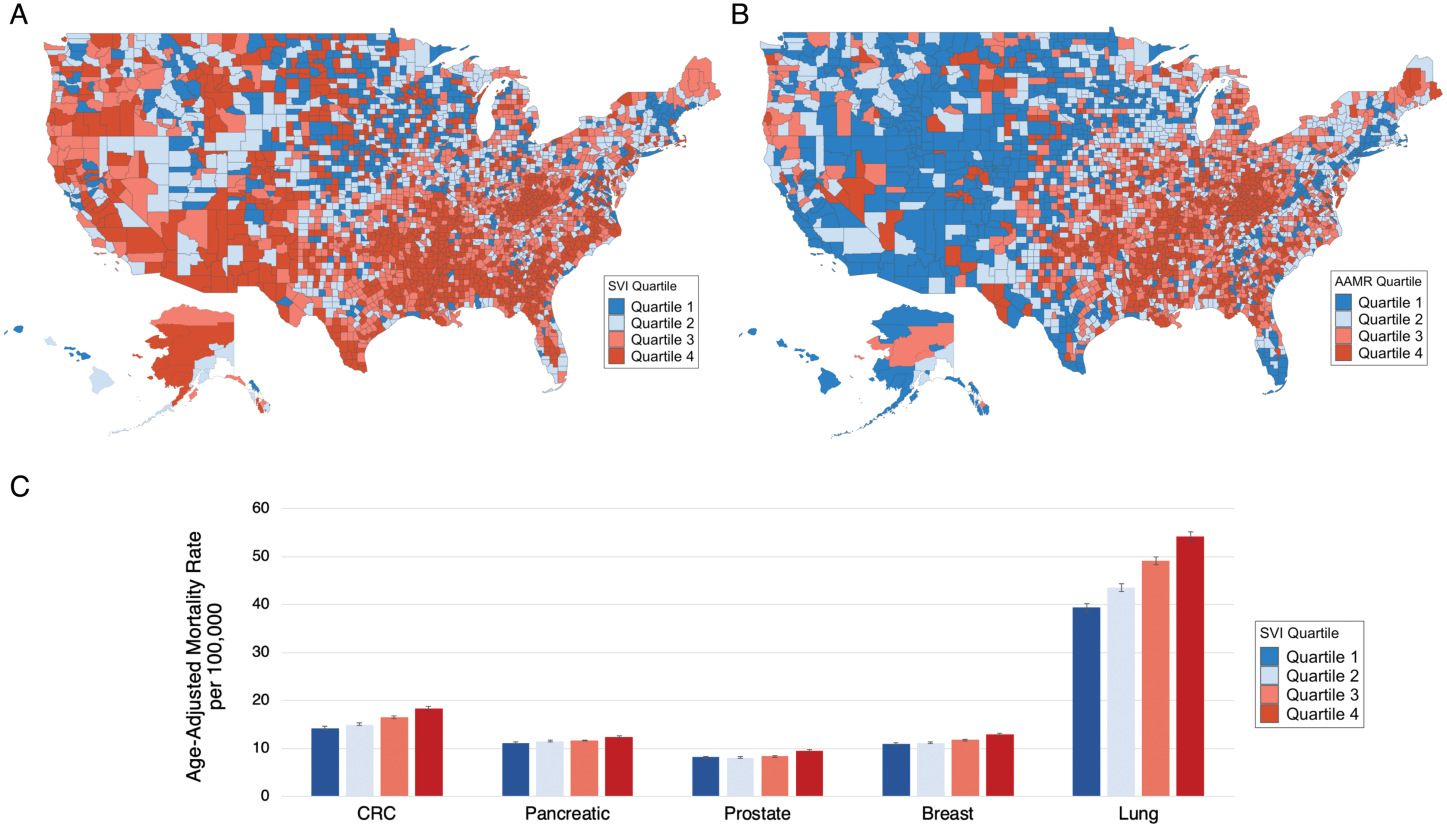
Social vulnerability index (SVI) and cancer-related age-adjusted mortality rate (AAMR) in the US, 2013 to 2019. A) US Counties by age-adjusted mortality rate per 100 000 adults for cancer, B) US counties by modified social vulnerability index quartile, C) Overall age-adjusted mortality increased linearly with increasing social vulnerability index for each of the 5 diseases that account for highest cancer deaths in the US. Error bars indicate the 95% CI for AAMR. CRC; colorectal cancer; SVI, Social Vulnerability Index.

There were 4 107 273 cancer-related deaths in the US between 2013 and 2019 with an overall AAMR of 173 per 100,000 individuals. Highest AAMRs were noted among older adults >65 years, men, non-Hispanic Black individuals, rural counties, and the Southern region. AAMRs increased proportionally when moving from least to most vulnerable counties (Fig. 1). The most vulnerable counties (4th SVI quartile) had higher AAMRs compared to the least vulnerable counties (1st SVI quartile) (RR 1.09, 95% CI [1.08, 1.10]). This was pronounced for ages 45-65 (RR 1.23, 95% CI [1.21, 1.25]), Hispanic individuals (RR 1.11, 95% CI [1.06, 1.16]), rural counties (RR 1.17, 95% CI [1.15, 1.19]), and Southern region (RR 1.17; 95% CI [1.16, 1.19]) (Table 1). When comparing most to least vulnerable counties among the separate SVI subcomponents, impact of vulnerable socioeconomic status was most notable in rural counties and Southern region, household barriers were most notable among ages 45-65 and the Southern region, and housing/transportation barriers were most notable among Hispanic and non-Hispanic Black individuals.

**Table 1.** Association between age-adjusted mortality rate (AAMR) per 100 000 and social vulnerability index (SVI).

	AAMR per 100 000 (95% CI)			Rate Ratio between 4th vs 1st SVI Quartile By Composite and SVI Subcomponent; RR (95% CI)			
	Total AAMR	1st SVI quartile	4th SVI quartile	Composite SVI	Socioeconomic status	Household	Housing/transportation
Overall	173.2 (172.2-174.1)	159.5 (157.6-161.4)	190.3 (188.2-192.4)	1.09 (1.08-1.10)	1.06 (1.05-1.07)	1.06 (1.05-1.07)	1.02 (1.01-1.03)
*Age*							
<45	11.5 (11.3-11.7)	10.2 (9.9-10.5)	13.9 (13.2-14.5)	1.11 (1.08-1.15)	1.07 (1.04-1.12)	0.97 (0.95-0.99)	1.08 (1.04-1.12)
45-65	202.3 (200.4-204.2)	170 (166.8-173.2)	242.4 (238.6-246.3)	1.23 (1.21-1.25)	1.16 (1.14-1.19)	1.13 (1.12-1.15)	1.12 (1.08-1.13)
>65	964.3 (959.6-969.0)	918.8 (908.2-929.5)	1018 (1007.6-1028.4)	1.05 (1.04-1.05)	1.03 (1.02-1.04)	1.03 (1.02-1.04)	1.00 (0.99-1.01)
*Sex*							
Men	209.7 (208.5-211.1)	191.5 (188.8-194.2)	234.3 (231.4-237.3)	1.11 (1.10-1.13)	1.08 (1.07-1.09)	1.06 (1.05-1.07)	1.03 (1.01-1.04)
Women	146.6 (145.8-147.5)	137.1 (135.4-138.7)	157.9 (156.1-159.6)	1.06 (1.05-1.07)	1.04 (1.03-1.05)	1.05 (1.04-1.06)	1.02 (1.01-1.03)
*Ethnicity/race*							
Hispanic	115.1 (112.4-117.9)	105.7 (99.9-111.5)	132.9 (124.8-140.9)	1.11 (1.06-1.16)	1.01 (0.96-1.05)	0.97 (0.94-1.00)	1.15 (1.10-1.23)
NH White	174.8 (173.8-175.7)	160.6 (158.7-162.5)	191.5 (189.4-193.6)	1.10 (1.09-1.11)	1.07 (1.06-1.08)	1.05 (1.04-1.06)	1.02 (1.01-1.03)
NH Black	194.5 (192.3-196.6)	176.2 (169.0-183.3)	207.4 (203.8-211.1)	1.11 (1.08-1.13)	1.06 (1.04-1.09)	1.08 (1.06-1.10)	1.13 (1.12-1.16)
*County type*							
Urban	168.8 (167.5-170.1)	160.1 (157.3-162.8)	183.9 (179.8-187.9)	1.03 (1.01-1.04)	0.99 (0.97-1.00)	1.01 (1.00-1.02)	1.01 (1.00-1.033)
Rural	175.9 (174.6-177.2)	159.1 (156.5-161.6)	192.2 (189.7-194.6)	1.17 (1.15-1.19)	1.178 (1.16-1.19)	1.12 (1.10-1.14)	1.05 (1.03-1.07)
*Region*							
Northeast	165.1 (163.0-167.2)	163 (159.6-166.4)	172.2 (158.0-186.3)	0.95 (0.93-0.98)	0.91 (0.89-0.93)	0.93 (0.91-0.95)	0.96 (0.91-1.00)
Midwest	170.8 (169.5-172.2)	161.3 (159.0-163.6)	195.6 (190.8-200.4)	0.99 (0.99-1.01)	0.96 (0.95-0.98)	1.03 (1.01-1.04)	0.99 (0.98-1.01)
South	181.7 (180.2-183.1)	160.4 (155.4-165.5)	193.7 (191.4-196.0)	1.17 (1.16-1.19)	1.20 (1.18-1.22)	1.17 (1.20-1.19)	1.06 (1.05-1.08)
West	149.7 (147.3-152.2)	142.5 (136.7-148.2)	162.3 (156.8-167.8)	1.09 (1.07-1.15)	1.08 (1.05-1.10)	1.01 (1.00-1.03)	1.07 (1.04-1.11)
*Cancer type*							
Colorectal	16.1 (16.0-16.2)	14.3 (14.0-14.6)	18.4 (18.0-18.7)	1.15 (1.13-1.17)	1.10 (1.08-1.12)	1.07 (1.05-1.08)	1.07 (1.04-1.09)
Pancreatic	11.7 (11.6-11.8)	11.2 (11.0-11.4)	12.4 (12.1-12.6)	1.02 (1.00-1.03)	1.01 (0.99-1.03)	1.03 (1.01-1.04)	1.01 (0.99-1.03)
Prostate	8.5 (8.4-8.6)	8.2 (8.0-8.4)	9.6 (9.3-9.8)	1.06 (1.04-1.09)	1.03 (1.01-1.06)	1.00 (0.98-1.02)	1.09 (1.06-1.12)
Breast	11.7 (11.6-11.8)	11.0 (10.7-11.2)	13.0 (12.7-13.2)	1.07 (1.05-1.09)	1.04 (1.02-1.06)	1.09 (1.00-1.03)	1.04 (1.02-1.07)
Lung	46.5 (46.1-47.0)	39.3 (38.5-40.2)	54.1 (53.1-55.1)	1.17 (1.15-1.19)	1.12 (1.09-1.14)	1.10 (1.08-1.12)	0.99 (0.97-1.02)

Abbreviations: AAMR, age-adjusted mortality rate; SVI, social vulnerability index; NH, non-Hispanic.

Five cancers with greatest mortality in the US comprised 51.9% of our cohort: lung (1,006,915; 24.5%), colorectal (354,756; 8.6%), pancreatic (286,795; 7.0%), breast (203,159; 4.9%), and prostate (203,159; 4.9%). While AAMR increased across all cancer types when moving from least to most vulnerable counties, greatest increases were noted in lung (RR 1.17, 95% CI [1.15, 1.19]) and colorectal cancer (RR 1.15, 95% CI [1.13, 1.17]). Impact of vulnerable socioeconomic status and household barriers was most prominent in lung and colorectal cancer, while housing/transportation was most prominent in prostate and colorectal cancer.

## Discussion

In this nationwide study, we demonstrate concerning trends where the most socially vulnerable US counties have higher cancer mortality rates than the least vulnerable US counties with significant sociodemographic and disease-site variation.

Rural populations and Southern regions were consistently impacted in all SVI subgroups, particularly socioeconomic and household-related disadvantages. Lower income, insurance rates, and educational attainment may potentially interact with a higher prevalence of adverse health-related behaviors in rural regions such as poor nutritional intake, sedentary lifestyle, and increased smoking, and contribute to higher cancer mortality.^5,6^ Rural populations, in particular, were likely also impacted by reduced access to local specialty care, transportation barriers and associated travel costs (including time, lost wages, child care demands, lodging).^5-7^ Indeed, recent evidence has noted a significant mortality divide between rural and urban regions, where rural regions have noted slower decline in mortality compared to urban counterparts, resulting in a widening disparity over 2 decades. This growing mortality divide is additionally accompanied by inequities in place of death, where rural residents die less often in a hospice facility.

Colorectal and lung cancer had higher mortality rates in socially vulnerable counties likely due to greater concentration of exposures in these areas.^6^ Moreover, these cancers are notable for their aggressive course, such that increased barriers to care and delays to diagnosis and treatment may exacerbate severity and mortality.^8,9^ Adults aged 45-65 may be particularly impacted by underutilization of healthcare relative to their risk as recommendations for cancer screening increase over this age range.

We situate these findings in the context of the new Justice40 initiative, led by the Biden-Harris administration to re-invest 40% of federal funding (including Health and Human Services, and the Department of Housing and Urban Development) in disadvantaged communities, as well as the reignition of the Cancer Moonshot initiative, which aims to reduce cancer-related deaths by 50%.^10^ As vulnerable counties bear greatest burdens in cancer mortality, our study provides a valuable metric in SVI that may assist state- and federal-level deliberations of invested funds in achieving geographic parity across socially disinvested counties.

Due to the cross-sectional nature of the study, we cannot establish causality or direction of association. Because some counties have no reported cancer deaths for certain subgroups, AAMR data for each subgroup represents a unique set of counties and cannot be directly compared across groups.

## Supplementary Material

oyad176_suppl_Supplementary_Tables

## Data Availability

The data underlying this article will be shared on reasonable request to the corresponding author.
