# Supplementary material for: Local Social Vulnerability as a Predictor for Cancer-Related Mortality Among US Counties
Source: Oncologist. 2023 Jun 19;28(9):e835–8. doi: 10.1093/oncolo/oyad176 (PMC10485383; doi:10.1093/oncolo/oyad176)
Supplement: oyad176_suppl_Supplementary_Tables [file oyad176_suppl_supplementary_tables.docx]

**Supplementary Table 1**: Social Determinants of Health Metrics in the Social Vulnerability Index

| **SVI Component** | **SVI Subcomponents** |
| --- | --- |
| SVI Component 1: Socioeconomic Status | Below Poverty |
|  | Unemployed |
|  | Income |
|  | No High School Diploma |
| SVI Component 2: Household Composition & Disability | Aged 65 or Older |
|  | Aged 17 or Younger |
|  | Civilian with a Disability |
|  | Single Parent Households |
| SVI Component 3: Minority Status & Language | Minority |
|  | Speaks English “Less than Well” |
| SVI Component 4: Housing Type & Transportation | Multi-Unit Structures |
|  | Mobile Homes |
|  | Crowding |
|  | No Vehicle |
|  | Group Quarters |

**Note**: Social Vulnerability Index by the CDC Agency for Toxic Substances and Disease Registry comprises 15 different social determinants of health metrics as noted above. SVI; Social Vulnerability Index

**Supplementary Table 2**: States included in US Regions

| **US Region** | **States** |
| --- | --- |
| Northeast | Connecticut, Maine, Massachusetts, New Hampshire, New Jersey, New York, Pennsylvania, Rhode Island, Vermont |
| Midwest | Illinois, Iowa, Kansas, Michigan, Minnesota, Missouri, Nebraska, North Dakota, Ohio, South Dakota, Wisconsin |
| South | Alabama, Arkansas, Delaware, District of Columbia, Florida, Georgia, Kentucky, Louisiana, Maryland, Mississippi, North Carolina, Oklahoma, South Carolina, Tennessee, Texas, Virginia, West Virginia |
| West | Alaska, Arizona, California, Colorado, Hawaii, Idaho, Montana, Nevada, New Mexico, Oregon, Utah, Washington, Wyoming |

**Note**: US geographic regions were defined by the CDC National Center for Health Statistics
